# The Risk of Latent Tuberculosis Infection Among Healthcare Workers at a General Hospital in Bisha, the Kingdom of Saudi Arabia

**DOI:** 10.7759/cureus.40561

**Published:** 2023-06-17

**Authors:** Naif S Alshahrani, Malik Kayal, Hawazen Alahmad Almshhad, Qais Dirar, Wael AlKattan, Atef Shibl, Abderrahman Ouban

**Affiliations:** 1 Immunology and Microbiology, Alfaisal University College of Medicine, Riyadh, SAU; 2 Anatomical Sciences, Alfaisal University College of Medicine, Riyadh, SAU; 3 Pathology, Alfaisal University College of Medicine, Riyadh, SAU; 4 Surgery, Alfaisal University College of Medicine, Riyadh, SAU

**Keywords:** rural health, risk factors, healthcare worker, latent, tuberculosis

## Abstract

Background and objective

Tuberculosis (TB) is a global health issue, often preceded by a latent tuberculosis infection (LTBI) in individuals. Significant global and local efforts have recently been directed toward this infection, focusing on TB control and eradication. This study aimed to assess the magnitude of LTBI among healthcare workers (HCWs) in the Kingdom of Saudi Arabia, by evaluating its prevalence and associated risk factors.

Methods

An analytical cross-sectional study was conducted among HCWs at the King Abdullah Hospital (KAH), from January to August 2018, by using two surveys: the first one involved data related to HCW demographics and the tuberculin skin test (TST) readings, and the second involved a questionnaire that assessed LTBI risk factors.

Results

Out of the total 561 HCWs who participated in the study, 66 had an induration reading of more than 10 mm in TST. Many factors were associated with LTBI cases, but multivariate analysis showed that age, gender, and nationality were statistically significant risk factors.

Conclusion

Given the nature of their work, HCWs are at a greater risk of TB and LTBI. At the same time, HCWs are uniquely positioned to play a crucial role in halting the spread of TB. Gaps in preventive measures may result in the increased spread of TB. Our study assessed risk factors associated with the increased risk of LTBI and proposed possible ways of addressing them.

## Introduction

Tuberculosis (TB) is a resurgent disease of major public health significance [1]. TB is currently the leading infectious disease cause of death globally, eclipsing malaria and HIV [2]. Fortunately, TB and latent tuberculosis infection (LTBI) can be treated and are preventable by following protective precautions in all risk groups. LTBI is defined as a condition where TB bacteria (Mycobacterium tuberculosis) is present with no signs or symptoms [3]. It is estimated that about 1% (approximately 55.5 million) of the global population has had recent LTBI [3]. In 2014, the global burden of LTBI was 23.0%, amounting to approximately 1.7 billion people. Western-Pacific, Southeast Asia, and African regions had the highest prevalence and accounted for around 80% of people with LTBI [3].

Healthcare workers (HCWs) from all specialties are at increased risk of TB due to the nature of their work [4]. The risk of contracting TB varies among different healthcare specialties; for instance, previous reports have indicated that HCWs who work in respiratory medicine wards carry a higher risk compared to those who work in pediatric wards [5]. Other essential factors to be considered include the age of the HCW and their work experience. Older age, waning immunity, and longer work history result in an increased likelihood of exposure to and a higher propensity to develop TB [6]. Gender, on the other hand, is a controversial risk factor, because, in some settings/occasions, males are more affected, while in others, the opposite is the case [4,6].

There is a high risk of TB transmission in Saudi Arabia, especially due to the high number of visitors during the annual Hajj season. Some of these visitors hail from TB-endemic countries [7,8]. Furthermore, recent political conflicts in our neighboring countries have affected the nearby rural areas in Saudi Arabia, including the Bisha region, and impacted their healthcare system leading to poor infection control measures [9]. There is a scarcity of comprehensive data regarding the magnitude of LTBI in Saudi Arabia. There are also wide variations in the prevalence rates reported among different healthcare facilities; while a tertiary hospital from the central region of the country has reported a prevalence rate of 31.5% among its staff [9], a clinical laboratory in the eastern region has reported a prevalence rate of 19.4% among its personnel [10].

## Materials and methods

After obtaining the IRB approval from the Institutional Ethics Committee (IRB#: 7220934), this cross-sectional study was conducted at the King Abdullah Hospital (KAH) in Bisha, Saudi Arabia, from January 2018 till August 2018. The inclusion criteria included all HCWs directly in contact with hospital patients (including physicians, nurses, and other technicians) or patient samples (i.e., laboratory HCWs). HCWs with positive tuberculin skin test (TST) results were clinically evaluated to rule out active TB, which would lead to exclusion in cases of confirmed disease. 

HCWs and professionals in KAH were screened for LTBI using TST, the standard LTBI screening test routinely employed in low- and middle-income countries. The Mantoux technique was used to administer the TST and measure the results, which involved injecting purified protein derivative (PPD) into the skin, followed by measuring the indurated margins within 48 hours [7]. A skin induration greater than 10 mm was considered a positive test. A negative test showed an induration of less than 10 mm in diameter, absence of reaction, or just erythema (red discoloration) of the skin.

Surveys addressing workers' demographics (age, gender, nationality, marital status, educational level, accommodation status, job, and work experience), potential risk factors (TB contact, smoking history, hospital admissions, comorbidities, and medications), and TST results were distributed among HCWs in the hospital. This step was preceded by a pilot study, which included 10 healthcare workers from outside the hospital, to assess the feasibility and applicability of the questionnaire-based survey.

Statistical analyses

Demographic data were collected, assessed, and then analyzed using IBM SPSS Statistics (IBM Corp., Armonk, NY) with a confidence interval (CI) of 95% to conduct descriptive analysis and the Chi-square test. All participants consented to be part of the study beforehand.

## Results

The study included 561 HCWs at KAH in Bisha who were evaluated from January 1 till the end of August 2018. Of them, 66 were found to have a positive TST result (Tables 1-4).

**Table 1:** Sociodemographic characteristics of healthcare workers at the King Abdullah Hospital in Bisha, 2018 SD: standard deviation

Sociodemographic characteristics		Frequency (n=561)	Percent (%)
Age group, years	<30	204	36.4
	30–39	181	32.3
	40–49	109	19.4
	≥50	67	11.9
	Range, mean ± SD	23–71, 36.08 ± 10.131	
Gender	Female	411	73.3
	Male	150	26.7
Nationality	Saudi	51	9.1
	Non-Saudi	510	90.9
Educational level	Bachelor's degree or below	404	72.0
	Higher than bachelor's	157	28.0
Residence	Outside hospital dorms	176	31.4
	Hospital dorm	385	

**Table 2:** Distribution of TST results according to sociodemographic characteristics among healthcare workers at the King Abdullah Hospital in Bisha, 2018 *Chi-square. ^♦^Fisher’s exact probability TST: tuberculin skin test

Sociodemographic characteristics		TST result		P-value*
		Negative, n=495, n (%)	Positive, n=66, n (%)	
Age group, years	<30	192 (94.1%)	12 (5.9%)	<0.001
	30–39	161 (89.0%)	20 (11.0%)	
	40–49	92 (84.4%)	17 (15.6%)	
	≥50	50 (74.6%)	17 (25.4%)	
Gender	Female	375 (91.2%)	36 (8.8%)	<0.001
	Male	120 (80%)	30 (20%)	
Nationality	Saudi	50 (98.0%)	1 (2.0%)	0.023^♦^
	Non-Saudi	445 (87.3%)	65 (12.7%)	
Residence	Outside hospital dorms	150 (85.2%)	26 (14.8%)	0.135
	Hospital dorm	345 (89.6%)	40 (10.4%)	
Educational level	Bachelor's or less	368 (91.1%)	36 (8.9%)	0.001
	Higher than bachelor's	127 (80.9%)	30 (19.1%)	

**Table 3:** Distribution of TST results according to certain work-related factors among healthcare workers at the King Abdullah Hospital in Bisha, 2018 *Chi-square. ^♦^Monte Carlo exact probability test ICU: intensive care unit; NICU: neonatal intensive care unit; TST: tuberculin skin test

Work-related factors		TST result		P-value*
		Negative, n=495, n (%)	Positive, n=66, n (%)	
Job	Doctors	133 (80.6%)	32 (19.4%)	0.002^♦^
	Nurses	336 (91.6%)	31 (8.4%)	
	Others	26 (89.7%)	3 (10.3%)	
Department	Emergency	52 (82.5%)	11 (17.5%)	0.404^♦^
	Medical	44 (88.0%)	6 (12.0%)	
	Surgical	88 (85.4%)	15 (14.6%)	
	Pediatric	15 (88.2%)	2 (11.8%)	
	OB/GYN	51 (85.0%)	9 (15.0%)	
	ICU	59 (90.8%)	6 (9.2%)	
	NICU	54 (87.1%)	8 (12.9%)	
	Lab	15 (88.2%)	2 (11.8%)	
	Others	117 (94.4%)	7 (5.6%)	
Working years	Less than 10	295 (92.5%)	24 (7.5%)	<0.001
	10 or more	200 (82.6%)	42 (17.4%)	

**Table 4:** Distribution of TST results according to medical history among healthcare workers at the King Abdullah Hospital in Bisha, 2018 *Chi-square. ^♦^Fisher's exact test TST: tuberculin skin test

Medical history		TST result		P-value*
		Negative, n=495, n (%)	Positive, n=66, n (%)	
Smoking history	No	453 (88.5%)	59 (11.5%)	0.566
	Yes	42 (85.7%)	7 (14.3%)	
Hospital admission	No	312 (88.6%)	40 (11.4%)	0.702
	Yes	183 (87.6%)	26 (12.4%)	
Chronic disease	No	461 (88.5%)	60 (11.5)	0.326^♦^
	Yes	34 (85.0%)	6 (15%)	
Surgical history	No	351 (89.8%)	40 (10.2%)	0.087
	Yes	144 (84.7%)	26 (15.3%)	
Long-standing medication	No	456 (88.2%)	61 (11.8%)	0.931
	Yes	39 (88.6%)	5 (11.4%)	
TB contact	No	284 (87.7%)	40 (12.3%)	0.618
	Yes	211 (89%)	26 (11%)	

Binary logistic regression using the Enter model for independent variables of TST results among HCWs at KAH in Bisha (as shown below in Table 5) shows the effect of each risk factor individually, taking into account all other factors. Nationality group, age, and gender were significant risk factors. This model shows that an increase in age by one year increased the risk of a positive TST by about 3%. In addition, males had almost double the risk of a positive TST than females. For nationality groups, non-Saudis were about 10 times more at risk of getting a positive TST compared to the Saudis. Moreover, the table shows that an increase in one year in work years is associated with a slight increase in risk (about 0.01%) of having a positive TST.

**Table 5:** Binary logistic regression using the Enter model for independent variables of TST results among healthcare workers at the King Abdulah Hospital in Bisha, 2018 B: beta coefficient; SE: standard error; Sig.: significance; OR: odds ratio; CI: confidence interval; TST: tuberculin skin test

Predictors	B	SE	Sig.	OR	95% CI for OR	
					Lower	Upper
Age	0.026	0.027	0.326	1.027	0.974	1.081
Gender	0.810	0.468	0.084	2.247	0.898	5.626
Nationality	2.347	1.110	0.034	10.456	1.187	92.111
Educational level	0.081	0.397	0.838	1.084	0.498	2.359
Job	0.443	0.392	0.258	1.558	0.723	3.357
Years of work	0.001	0.029	0.960	1.001	0.946	1.060
TB policy reading	-0.427	0.321	0.183	0.653	0.348	1.223
Constant	-6.848	1.792	0.000	0.001		
Model pseudo R^2^ (p-value)			10.5% (p<0.001)			
Model accuracy			88.2%			

Binary logistic regression using the stepwise model for independent variables of TST results among HCWs at KAH in Bisha (as shown below in Table 6) highlights the most critical risk factors associated with TST results after removing all other non-significant factors. Age, gender, and nationality were significant risk factors. It is clear from the table that an increase in age by one year is associated with an increased risk of getting a positive TST by about 3% and males had almost double the risk of getting a positive TST than females, as shown in the previous model. Regarding nationality, non-Saudis were about seven times more at risk of getting a positive TST compared to the Saudis.

**Table 6:** Binary logistic regression using backward stepwise (likelihood ratio) model for independent variables of TST results among healthcare workers at King Abdulah Hospital in Bisha, 2018 B: beta coefficient; SE: standard error; Sig.: significance; OR: odds ratio; CI: confidence interval; TST: tuberculin skin test

Predictors	B	SE	Sig.	OR	95% CI for OR	
					Lower	Upper
Age	0.029	0.014	4.213	1.030	1.001	1.059
Gender	0.728	0.328	4.926	2.072	1.089	3.941
Nationality	1.979	1.038	3.637	7.235	0.947	55.298
Constant	-5.265	1.081	23.720	0.005		
Model pseudo R^2^ (p-value)			9.2% (p<0.001)			
Model accuracy			88.2%			

The awareness survey involving HCWs at the hospital included several questions regarding important LTBI and TST-related issues. These questions were included to assess the level of awareness and understanding among HCWs. Analysis of the results of this survey revealed that almost half of the HCWs were not aware of the difference between TB and LTBI or the importance of LTBI treatment (42.6% and 42.2%, respectively). Moreover, more than three-quarters of the HCWs were not aware of the purpose of TST.

## Discussion

This study aimed to assess the risk of LTBI among HCWs at KAH in Bisha. Recently published guidelines for the prevention of TB transmission in healthcare settings include baseline (preplacement) screening and testing, post-exposure screening and testing, and serial screening and testing for healthcare personnel without LTBI, as well as evaluation and treatment of healthcare personnel with positive test results [11]. In our study, TST was conducted among HCWs of this hospital to be assessed for potential LTBI risk factors. The survey involved the first comprehensive screening to be conducted among HCWs in this hospital. It included all new and old healthcare workers, targeting new and old TB infections, which caused difficulty in knowing about real-time infection.

The results revealed that males were more likely to get a positive TST than females. While this finding is in line with some studies in the literature [5,6], it contrasts with the findings of some other studies revealing a preponderance of TBI among female HCWs [9,12,13], as in the study by Hsieh et al., which reported no correlation between gender and increased prevalence of LTBI [14]. One theory suggests that males are more mobile and have more contacts outside the hospital than females. This finding corresponds to the epidemiological distribution of TB among genders worldwide [1]. In addition, the results of our study indicated that the incidence of LTBI was highest in the age group of 50 years and over, and an increase in age by one year is associated with an increased risk of getting a positive TST by about 3%, which agrees with other recent findings in the literature [3,13].

This study showed a significantly greater LTBI prevalence among non-Saudis compared to Saudis, which concurs with many local studies [9]. This could be explained by the higher prevalence of TB in their native countries such as Nigeria, the Philippines, Pakistan, India, and Bangladesh [1].

In our study, the prevalence of LTBI among physicians outweighed that in other HCWs. By contrast, one study has reported that the prevalence was highest in nurses among HCWs [13]. Another study has reported that the type of healthcare occupation did not correlate with an increase in the prevalence of LTBI [14]. Our findings revealed a higher prevalence of LTBI in HCWs with higher education (defined as having a degree higher than a bachelor's), which is similar to the findings of other studies in the literature [15]. One explanation for this is that those with higher degrees had graduated from older medical schools that may lack infection control practices, leading to an underestimation of risk and lack of awareness among these HCWs.

Our results indicate that about 11.8% of HCWs (66/561) had a positive TST result, with regard to LTBI [7], which is slightly higher compared to the findings of another study conducted in Saudi Arabia [15]. However, our results are lower compared to a meta-analysis involving 46 non-Saudi hospitals, which may be as high as 62% [16].

While the scope of the study did not include the overall prevalence of TB in the hospital, it is important to assess this metric given that measures have been instituted to prevent the spread of LTBI [5]. High prevalence among patients, which can lead to a higher prevalence of occupational TB [if personal protective equipment (PPE) is used, HCWs are not supposed to be infected despite the high prevalence in patients], and may undermine HCWs' contribution to the efforts to eliminate TB globally [17].

One aspect that should be considered is the psychological and emotional impact of LTBI diagnosis on HCWs, and the presence of any support systems in place to address these concerns [18-20]. The most common cause of stigma in this context is the perceived risk of transmission of the disease and TB’s association with malnutrition, poverty, and HIV infection [19,20]. While HCWs have better access to healthcare facilities than other societal groups, they will be reluctant to use them for fear of being stigmatized [21]. Addressing this stigma may have an overall positive impact on managing occupational TB [22,23], as prompt diagnosis and management will prevent its progression to active TB [18-23]. The physical and mental well-being of HCWs is of prime importance because of their unique role as advocates and effectors of change to raise awareness of and implement TB infection control measures [24].

Limitations

This study has a few limitations, primarily the fact that it was conducted at a single institution and confined to one region in the Arabian peninsula. In addition, the study was limited in terms of its time frame: January-August 2018. Also, there was no evaluation of the use of PPE among HCWs.

## Conclusions

We examined the prevalence of latent TB among HCWs at a Saudi Arabian hospital and correlated this with several social, demographical, and clinical parameters. Although the prevalence of LTBI was relatively low, gaps in preventive measures seen among HCWs in the hospital might affect the overall prevention process and jeopardize the control measures.

Our results highlight a pressing need to increase the depth and breadth of education on LTBI, TB, preventive control measures, and TB treatment among all HCWs. Incorporating TB and measures to control it into the curricula across all health colleges is of prime importance, given that TB, like many other infectious diseases, is on the rise. Using the lessons learned from the coronavirus disease 2019 (COVID-19) pandemic may prove vital in tackling many infectious diseases, including TB. Preventing crowding, using high-grade respirator face-masks, and early work-up of suspected cases may help in stemming the tide of infectious TB. Finally, addressing the stigma associated with TB infection among HCWs should be a priority, given that they are at the forefront of the fight against the disease.
